# Cholecystectomy and the risk of colorectal cancer in Italy

**DOI:** 10.1038/sj.bjc.6601721

**Published:** 2004-03-16

**Authors:** A Altieri, C Pelucchi, R Talamini, C Bosetti, S Franceschi, C La Vecchia

**Affiliations:** 1 Istituto di Ricerche Farmacologiche ‘Mario Negri’, via Eritrea 62, 20157 Milano, Italy; 2 Servizio di Epidemiologia e Biostatistica, Centro di Riferimento Oncologico, via Pedemontana Occ., 33081 Aviano (PN), Italy; 3 International Agency for Research on Cancer, 150 Cours Albert Thomas, 69372 Lyon Cedex 08, France; 4 Istituto di Statistica Medica e Biometria, Università degli Studi di Milano, via Venezian 1, 20133 Milano, Italy

**Keywords:** colorectal cancer, gallstones, cholecystectomy, cholelithiasis, risk factors

## Abstract

In two case–control studies from Italy covering 3533 cases of colorectal cancer and 7062 hospital controls, the odds ratios were 1.04 after cholecystectomy for colorectal, 1.08 for colon and 1.03 for rectal cancers. The results did not differ significantly by gender, colon subsite or time since diagnosis.

At least four large record linkage and cohort studies (Ekbom *et al*, 1993; Goldbohm *et al*, 1993; Johansen *et al*, 1996; Lagergren *et al*, 2001) and two meta-analyses (Giovannucci *et al*, 1993; Reid *et al*, 1996) found some increase of colorectal cancer risk following cholecystectomy. No association was found in another population-based cohort study from Sweden, including 150 colorectal cancer cases (Adami *et al*, 1987). The strength of the association, generally modest but significant in several studies (Ekbom *et al*, 1993; Giovannucci *et al*, 1993; Reid *et al*, 1996; Lagergren *et al*, 2001), has varied across different studies, colorectal subsites and sex (being somewhat stronger for proximal colon and for women). However different potential confounding factors and time since cholecystectomy have not always adequately been taken into account. Overweight and obesity are relevant risk factors for both gall bladder (Lew and Garfinkel, 1979; La Vecchia *et al*, 1991) and colorectal cancer (Lew and Garfinkel, 1979; Russo *et al*, 1998; Terry *et al*, 2001; Adami and Trichopoulos, 2003; Calle *et al*, 2003), and may therefore represent both an underlying pathogenetic mechanism and a potential confounding factor.

In the Nurses' Health Study, including 877 women diagnosed with colorectal cancer, an increased relative risk (RR) of 1.21 (95% confidence interval (CI) 1.01–1.46) was found in relation to history of gallstones or cholecystectomy (reported by 133 colorectal cancer cases), after adjusting for potential confounding factors (Schernhammer *et al*, 2003). The highest risks were reported for proximal colon (RR 1.34, 95% CI 0.97–1.88) and rectum cancer (RR 1.58, 95% CI 1.05–2.36).

We have examined the relation between gallstones and colorectal cancer risk using data derived from two large Italian case–control studies on colorectal cancer, taking advantage of information on body size and of a comprehensive food-consumption questionnaire.

## MATERIALS AND METHODS

The first study was conducted between 1985 and 1991 in the greater Milan area (La Vecchia *et al*, 1988; Ferraroni *et al*, 1994), and the second one between 1991 and 1996 in six Italian areas: Greater Milan, the province of Pordenone, the urban area of Genoa and the province of Forlì, in northern Italy; the province of Latina, in central Italy; and the urban area of Naples, in southern Italy (La Vecchia *et al*, 1997). Overall, there were 3533 patients, aged 19–79 years (median age 62) with incident (i.e. diagnosed within the year before interview), histologically confirmed cancer of the colon (2180 cases) or rectum (1353 cases), from the major teaching and general hospitals in areas under surveillance. Cancers of the colon and rectum and their anatomical subsites were defined according to the International Classification of Diseases, Ninth Edition (ICD-9). Colon cancer corresponded to ICD-9 153; ascending colon included ICD-9 153.0, 153.4, 153.5 and 153.6; transverse and descending colon ICD-9 153.1, 153.2 and 153.7; sigmoid colon corresponded to ICD-9 153.3. Rectosigmoid junction corresponded to ICD-9 154.0 and rectum to ICD-9 154.1.

Controls were 7062 patients, aged 19–79 years (median age 57), residing in the same geographical areas and from the same network of hospitals where cases had been identified, and admitted for a wide spectrum of acute, non-neoplastic conditions, unrelated to known or potential risk factors for colorectal cancer (30% traumas, mostly fractures and sprains; 25% nontraumatic orthopaedic conditions, mostly low back pain and disk disorders; 21% acute surgical diseases, mostly abdominal, such as acute appendicitis or strangulated hernia, and 24% other miscellaneous disorders, such as eye, ear, nose and throat and dental disorders). Less than 4% of cases and controls approached refused to participate. Information was collected in hospital by trained interviewers using a structured food-frequency questionnaire, tested for reproducibility and validity (Decarli *et al*, 1996). The patients were asked if they had a diagnosis of selected medical conditions, and the age at first diagnosis was recorded.

Odds ratios (OR) and corresponding 95% CIs were estimated using unconditional multiple logistic regression models, including terms for age (<35, 35–39, 40–44, 45–49, 50–54, 55–59, 60–64, 65–69, 70–74, ⩾75 years), sex, study centre, years of education (<7, 7–10, >10 years), cigarette smoking (never smokers, ex-smokers, smokers of <15, 15–24, ⩾25 cigarettes/day), alcohol drinking and meat consumption (tertiles of consumption), body mass index (BMI, kg/m^2^) and total energy intake (tertiles), history of diabetes, history of colorectal cancer in parents and siblings, menopausal status (pre-, postmenopause), use of oral contraceptives or hormone replacement therapy (ever, never use).

## RESULTS

Table 1

**Table 1:** Relation between history and time since diagnosis of cholelithiasis and colorectal cancer, among 3533 cases and 7062 controls (Italy, 1985-1996)

	**Men**		**Women**		**Total**	
**Risk factors**	**Cases : Controls**	**OR (95% CI)^a^**	**Cases : Controls**	**OR (95% CI)^a^**	**Cases : Controls**	**OR (95% CI)^a^**
*Colorectal cancer*						
History of cholelithiasis						
No	1858 : 3761	1^b^	1325 : 2696	1^b^	3183 : 6457	1^b^
Yes	139 : 191	1.21 (0.95–1.54)	211 : 414	0.95 (0.79–1.15)	350 : 605	1.04 (0.90–1.21)
Time since diagnosis (years)^c^						
<10	65 : 83	1.35 (0.95–1.92)	74 : 144	0.97 (0.72–1.32)	139 : 227	1.13 (0.90–1.42)
⩾10	72 : 107	1.11 (0.81–1.52)	137 : 270	0.96 (0.77–1.21)	209 : 377	0.99 (0.82–1.19)
						
*Colon cancer*						
History of cholelithiasis						
No	1103 : 3761	1^b^	854 : 2696	1^b^	1957 : 6457	1^b^
Yes	83 : 191	1.24 (0.93–1.64)	140 : 414	1.01 (0.81–1.26)	223 : 605	1.08 (0.91–1.28)
Time since diagnosis (years)^c^						
<10	41 : 83	1.47 (0.98–2.20)	47 : 144	0.95 (0.67–1.36)	88 : 227	1.16 (0.89–1.51)
⩾10	40 : 107	1.03 (0.70–1.52)	93 : 270	1.05 (0.80–1.36)	133 : 377	1.02 (0.82–1.26)
						
*Rectal cancer*						
History of cholelithiasis						
No	755 : 3761	1^b^	471 : 2696	1^b^	1226 : 6457	1^b^
Yes	56 : 191	1.22 (0.88–1.69)	71 : 414	0.93 (0.70–1.23)	127 : 605	1.03 (0.83–1.27)
Time since diagnosis (years)^c^						
<10	24 : 83	1.21 (0.75–1.97)	27 : 144	1.03 (0.66–1.59)	51 : 227	1.12 (0.81–1.54)
⩾10	32 : 107	1.25 (0.82–1.90)	44 : 270	0.88 (0.62–1.25)	76 : 377	0.98 (0.75–1.28)

a Estimates from unconditional multiple logistic regression models, including terms for age, sex, study centre, education, cigarette smoking, alcohol drinking, BMI, meat consumption, total energy intake, history of diabetes, history of colorectal cancer in parents and siblings, menopausal status, use of oral contraceptives or hormone replacement therapy.

b Reference category.

c The sum does not correspond to the total number of subjects because of some missing values.

shows the distribution of colorectal cancer cases and controls, and the corresponding ORs, according to history and time since diagnosis of cholelithiasis. Subjects with a history of cholelithiasis showed no appreciable increased risk of colorectal cancer (OR 1.04, 95% CI 0.90–1.21). Similar results were found for colon (OR 1.08, 95% CI 0.91–1.28) and rectal (OR 1.03, 95% CI 0.83–1.27) cancer. The OR was 1.03 (95% CI 0.72–1.47) for proximal colon and 1.17 (95% CI 0.93–1.47) for distal colon cancer. The ORs for men were 1.21 for colorectal, 1.24 for colon and 1.22 for rectal cancer, and for women were 0.95, 1.01 and 0.93 respectively. With reference to time since cholelithiasis, ORs for less than 10 years were 1.13 for colorectal, 1.16 for colon and 1.12 for rectal cancer; no association was found for 10 or more years before interview.

## DISCUSSION

Our findings indicate that cholelithiasis is not materially associated with colorectal cancer risk after adjustment for major identified confounding factors, including measures of BMI, total energy and meat intake. A modest nonsignificant excess risk was found among men and for less than 10 years after cholelithiasis, but no excess risk could be found after 10 or more years. Such a time-risk relation is inconsistent with a causal relation of cholelithiasis with colorectal cancer risk. The risk estimates were not heterogeneous for men and women. However, the finding that several ORs were apparently higher in men is in broad agreement with the observation, in the same data set, that higher BMI is more consistently related to colorectal cancer risk in men than in women (Russo *et al*, 1998). None of these estimates was significant, suggesting that any association is unlikely to be causal. No material association was observed when the analysis was restricted to proximal colon.

The apparent association reported from some case–control studies may at least in part be due to a more accurate recall of gall bladder disease by colorectal cancer patients and by the inadequate adjustment for other potential risk factors for colorectal cancer, including diet. In our study, based on a notably large data set, information on medical history was satisfactorily reproducible (Bosetti *et al*, 2001), indicating that recall bias is unlikely to have played a major role. Other potential biases of this study should be limited, given the almost complete response rate, the similar catchment area for cases and controls and the administration of a standard questionnaire under similar conditions.
